# Supplementary material for: A Novel Method That Allows SNP Discrimination with 160:1 Ratio for Biosensors Based on DNA-DNA Hybridization
Source: Biosensors (Basel). 2021 Aug 6;11(8):265. doi: 10.3390/bios11080265 (PMC8391390; doi:10.3390/bios11080265)
Supplement: Supplementary file 1 [file biosensors-11-00265-s001.zip › biosensors-1304733-supplementary.pdf]

A Novel Method that allows SNP Discrimination with 160:1 ratio for Biosensors Based on DNA-DNA hybridization

Satish Balasaheb Nimse,[a†] Keum-Soo Song,[b†] Shrikant Dashrath Warkad,[b] Taisun Kim[a*]

[a]Institute for Applied Chemistry and Department of Chemistry, Hallym University, Chuncheon, 200-702, Korea, Fax: +82-33-256-3421, E-mail: tskim@hallym.ac.kr

[b]Biometrix Technology, Inc. 202 BioVenture Plaza, Chuncheon, 200-161, Korea

Supporting Information

Table S1. Sequences of the probes used for derivation of SWAT

Probes	Sequence	T_m (ºC)	
		[4(G+C)+2(A+T)–5]	Nearest neighbor method
**Probe1**	5'-9G-vertical spacer-TAC CGA CCC ACG CGG GC-3'	55	55.96
**Probe2**	5'-9G-vertical spacer-TAC CGG CCC ACG CGG GC-3'	57	58.45
**Probe3**	5'-9G-vertical spacer-TAC CGG CCC ACC CGG GC-3'	57	56.30
**Probe4**	5'-9G-vertical spacer-TAC CGA CCC ACG TGG GC-3'	53	52.37
**Probe5**	5'-9G-vertical spacer-TAC CGG CCT ACG CGG GC-3'	55	56.78
**Probe6**	5'-9G-vertical spacer-TAC CGG CCC ACC TGG GC-3'	55	54.3

9G- nine consecutive guanines (GGG GGG GGG) used for the immobilization of the probes on the AMCA DNAChip; Vertical spacer- nine consecutive thymidine's (TTT TTT TTT); T_M: melting temperature

Table S2. Sequences of probes used for the detection and discrimination of MTB strains and NTM Strains

Probes	Probe Description	Sequence	T_m(ºC)
**Probe7**	*MTB*	5'-9G-vertical spacer-TAG CGA CCG ACG TGG GC-3'	53
**Probe8**	*Chelonae*	5'-9G-vertical spacer-TAC CGG CCT ACG CGG GC-3'	55
**Probe9**	*Avium*	5'-9G-vertical spacer-TAG CGG CCC ACC TGG GC-3'	55
**Probe10**	*Marinum*	5'-9G-vertical spacer-TAC CGG CCC ACC TGG GC-3'	55
**Probe11**	*Abscessus*	5'-9G-vertical spacer-TAC CGA CCC ACG TGG GC-3'	53
**Probe12**	*kansasii*	5'-9G-vertical spacer-TAT CGG CCG ACATGG GC-3'	53
**Probe13**	PCR	5'-9G-vertical spacer-CGA CCS ACV CGS GCC AGG TC -3'	61
**Probe14**	NC	5'-9G-vertical spacer-GCC AGG TCG TAG CGC TTC TC-3'	61
**Probe15**	HC	5'-9G-vertical spacer-CCT AGT GGC TCT ATG GTA AC-3'	55
**HC-Cy5-T1**	HC Target DNA	3'- GGA TCA CCG AGA TAC CAT TG GAG ACT GCG -Cy5-5'	

HC – probe for the Hybridization control; HC-Cy5-T1 – Target oligonucleotide for HC probe9G- nine consecutive guanines (GGG GGG GGG) used for the immobilization of the probes on the AMCA DNAChip; Vertical spacer- nine consecutive thymidine's (TTT TTT TTT); T_M: melting temperature

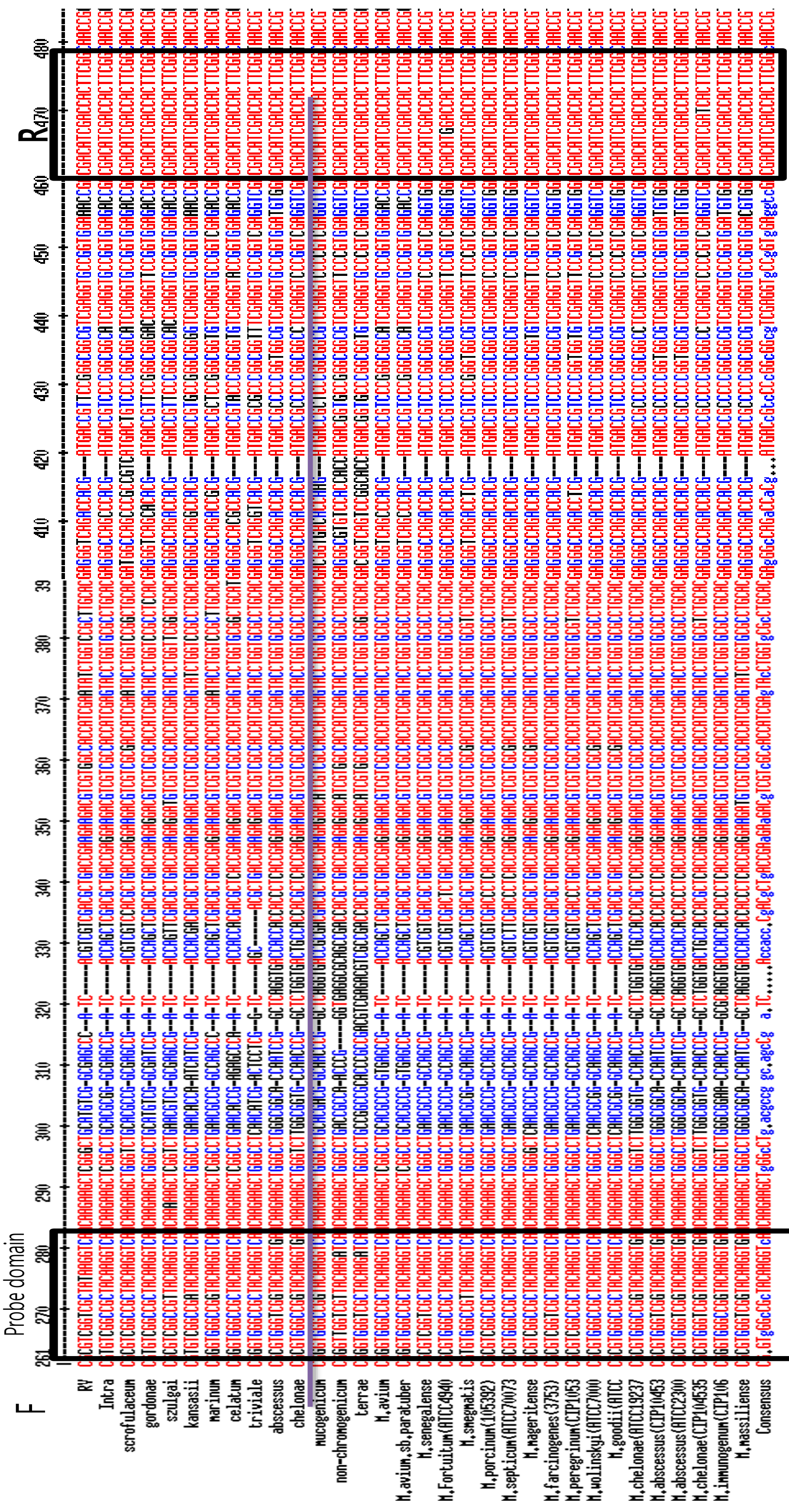

Figure S1: Homology in the sequences of the genomic DNA of the MTB and NTM strains

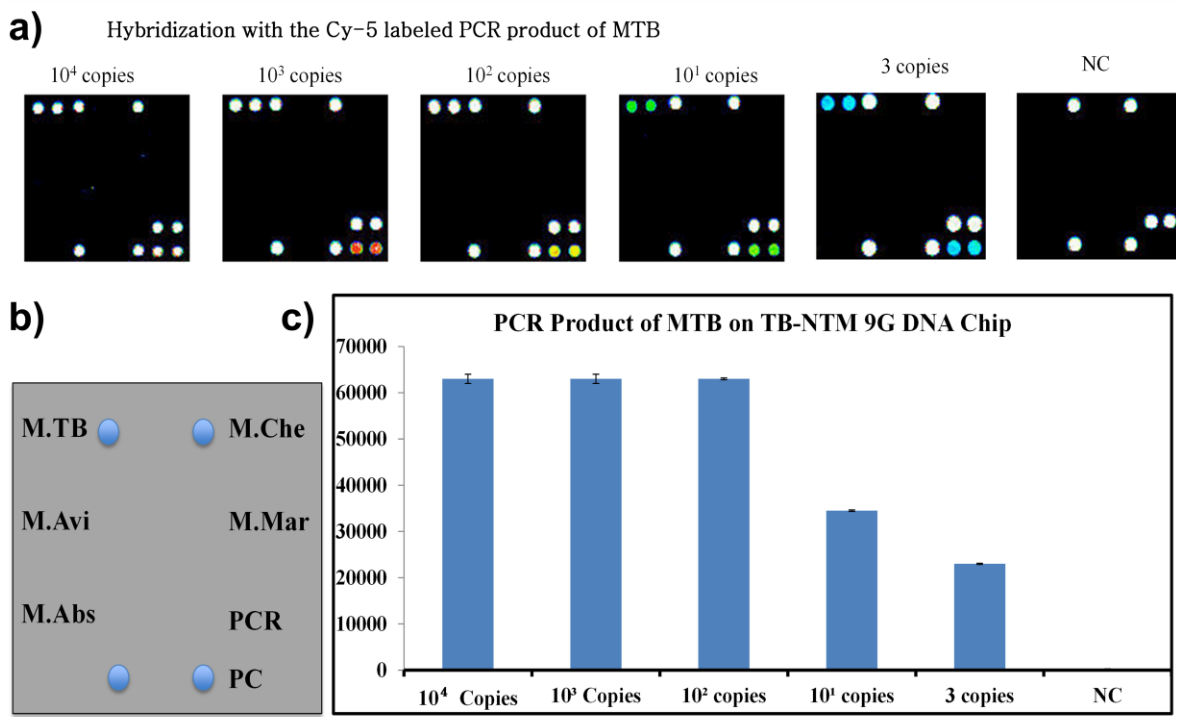

Figure S2: Limit of detection for the identification of MTB.

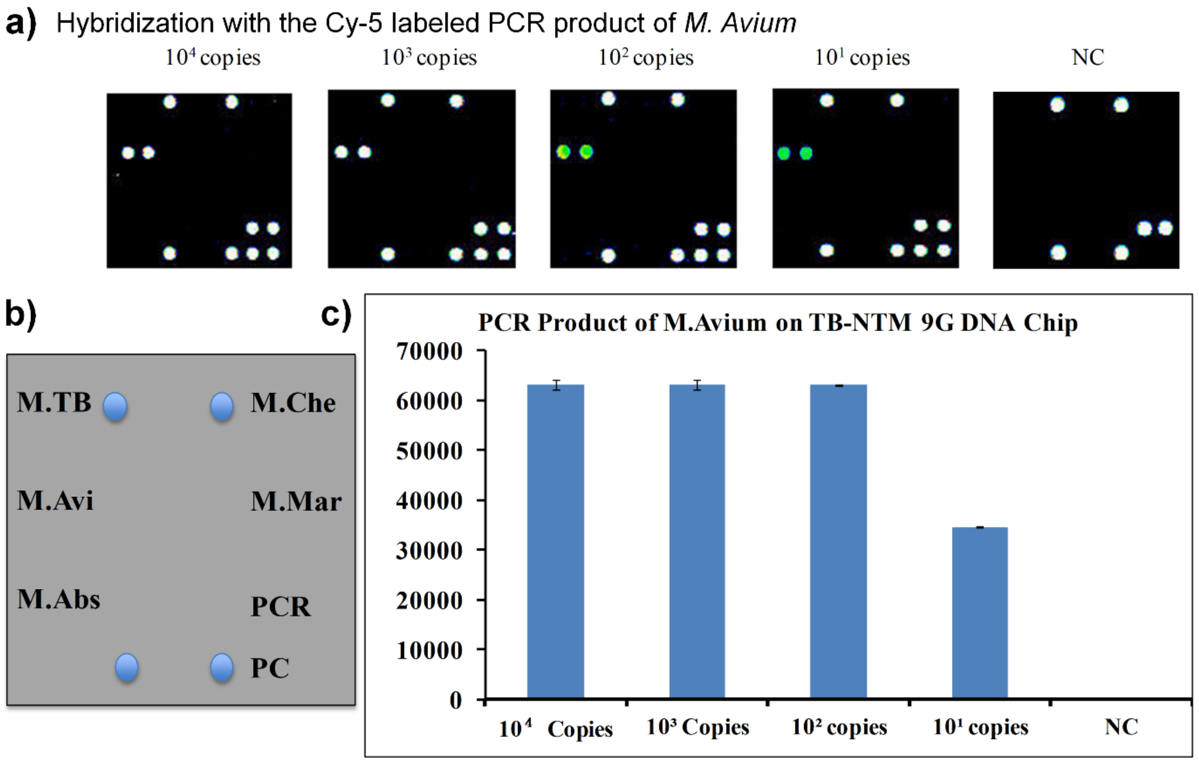

Figure S3: Limit of detection for the identification of *Mycobacterium Avium*.

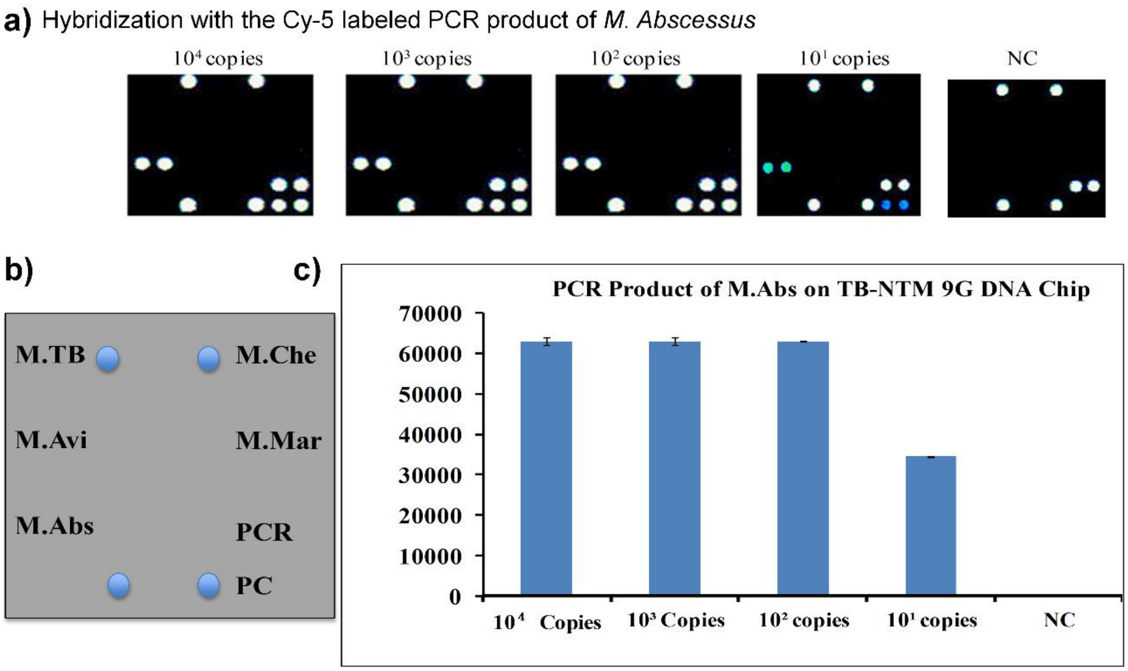

Figure S4: Limit of detection for the identification of *Mycobacterium Abscessus*.

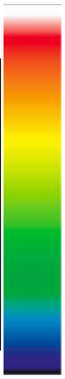

**Sample No.**	Sample 1	Sample 2	Sample 3
**Results**	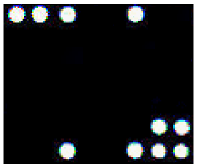	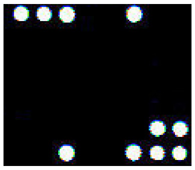	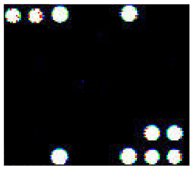
**Identified**	M.Tuberculosis	M.Tuberculosis	M.Tuberculosis

**Sample No.**	Sample 4	Sample 5	Sample 6	Sample 7
**Results**	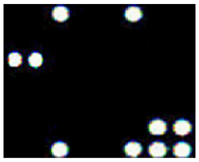	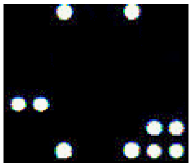	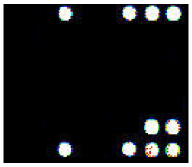	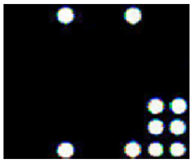
**Identified**	M.Avium	M.Abscessus	M.Chelonane	M.Kansassi

Figure S5: The detection and discrimination of the MTB and NTM strain in the clinical samples.
